# Intrahepatic cholestasis of pregnancy associated with azathioprine: first quantitative disproportionality analysis using the FDA adverse event reporting system

**DOI:** 10.3389/jpps.2025.15527

**Published:** 2025-12-16

**Authors:** Yonghoon Kwon, Nai Lee, Yun Kim

**Affiliations:** College of Pharmacy, Daegu Catholic University, Gyeongsan, Republic of Korea

**Keywords:** azathioprine, intrahepatic cholestasis of pregnancy, pregnancy safety, pharmacovigilance, signals of disproportionate reporting

## Abstract

**Introduction:**

Azathioprine (AZA) is an immunosuppressant approved for renal transplant rejection and rheumatoid arthritis. Recent FDA alerts have raised concerns about its link to intrahepatic cholestasis of pregnancy (ICP), a condition with serious maternal and fetal risks. This study used disproportionality analysis as a hypothesis-generating approach to evaluate the reporting association between AZA and ICP during pregnancy and to compare AZA with other drugs previously implicated in ICP.

**Methods:**

A retrospective pharmacovigilance study was conducted using the FDA Adverse Event Reporting System (FAERS) reports from 1968 to Q2 2024. Disproportionality analysis was performed using reporting odds ratios (RORs), with statistical significance defined as a lower limit of the 95% confidence interval (CI) >1 and at least three unique cases. Subgroup analyses were conducted by pregnancy status and underlying autoimmune indications, and comparative analyses were performed against drugs previously reported to induce ICP.

**Results:**

Among 35,576 AZA-related reports, 67 specifically documented ICP. A strong signal was detected for ICP ROR025 = 153.0; IC025 = 5.8; EBGM05 = 144.37), ranking among the highest AZA-associated adverse events. In pregnant women, ICP also showed a significant signal (ROR025 = 5.46; IC025 = 1.93; EBGM05 = 5.31). Subgroup analyses by indication revealed elevated risks in Crohn’s disease (ROR025 = 66.99; IC025 = 4.8; EBGM05 = 64.73), and Colitis ulcerative (ROR025 = 9.01; IC025 = 1.95; EBGM05 = 9.95). Comparative analyses demonstrated that AZA had a higher proportion of ICP cases than other drugs reported to induce ICP.

**Conclusion:**

This pharmacovigilance analysis identifies a disproportionality signal suggesting a possible association between AZA and intrahepatic cholestasis of pregnancy. These hypothesis-generating findings underscore the importance of cautious use and clinical vigilance when prescribing AZA to women of reproductive age.

## Introduction

The thiopurine class of immunosuppressant includes azathioprine (AZA), 6-mercaptopurine (MP), and thioguanine [1]. These agents are widely used in clinical practice to manage immune-mediated responses in organ transplantation and autoimmune disorders [2]. The pharmacological mechanism of thiopurines is well established: AZA is converted to thioguanine nucleotides (TGNs) by several enzymes [3–5]. TGNs consist of thioguanine monophosphate, thioguanine diphosphate and thioguanine triphosphate [6]. These active metabolites exert cytotoxic and immunosuppressive effects through multiple pathways, ultimately leading to T-lymphocyte suppression and apoptosis [7]. Among the three agents, AZA has the longest history of regulatory approval and is indicated by the U.S. Food and Drug Administration (FDA) for preventing rejection in renal transplantation and for managing active rheumatoid arthritis by reducing signs and symptoms [8]. In addition, AZA and other thiopurines are frequently used off-label for chronic inflammatory diseases such as Crohn’s disease (CD), ulcerative colitis (UC) and systemic lupus erythematosus (SLE) [9, 10].

Despite its therapeutic benefits, AZA is associated with several adverse events (AEs), including hepatotoxicity, malignancy, cytopenias and other serious infections [8, 11]. Among the various AEs, intrahepatic cholestasis of pregnancy (ICP) has emerged as a clinically relevant safety concern in pregnant women [12]. ICP is a liver disorder characterized by impaired bile flow and elevated serum bile acid levels, which are associated with adverse maternal outcomes and increased risk of preterm delivery, fetal distress, and stillbirth [13]. Although the exact etiology of ICP remains unclear, environmental, genetic, immunological, and hormonal factors are implicated [14]. Clinical manifestations of ICP may include pruritus, jaundice, right upper quadrant pain, nausea, poor appetite, or sleep disturbance [15]. According to FDA prescribing information, administration of AZA is not recommended during pregnancy [8]. However, treatment guidelines from the American College of Rheumatology and the American Gastroenterological Association suggest that continuation of AZA or MP may be appropriate in selected cases to manage underlying disease activity during pregnancy [16–19]. Therefore, careful monitoring for AZA-associated ICP is clinically important. AZA has been reported to induce transient elevations in liver enzyme levels and biochemical cholestasis [20].

Recent FDA safety communications have highlighted the potential risk of ICP associated with thiopurines, including AZA, thereby raising concerns about its safety profile in pregnant women [16]. While the current FDA prescribing information for AZA acknowledges the possibility of ICP and advises discontinuation upon diagnosis, it does not provide quantitative evidence to establish the strength of this association [8]. Furthermore, there is a lack of pharmacovigilance studies that systematically evaluate the relationship between AZA exposure and ICP during pregnancy. To our knowledge, the present work is the first quantitative pharmacovigilance analysis to assess the association between AZA and ICP using the U.S. FDA Adverse Event Reporting System (FAERS) database. In addition, we compared the disproportionality of AZA with other drugs known to cause ICP, thereby contextualizing the potential risk in pregnant women.

## Methods

### Data source: the FDA adverse event reporting system (FAERS)

The pharmacovigilance database of the FAERS was utilized for this study. The dataset includes patient demographics, drug exposure information, indication for drug use, reported AEs, and clinical outcomes [21]. All AEs in FAERS are coded using the Medical Dictionary for Regulatory Activities (MedDRA), an internationally standardized terminology widely employed by regulatory agencies and the pharmaceutical industry. The data structure of FAERS follows the International Council for Harmonization (ICH) E2B global safety reporting criteria.

### Case identification, data cleaning, and deduplication procedures

A retrospective analysis was conducted on ICP-related AEs associated with AZA reported in FAERS from 1968 through Q2 2024. In the demographic summary for AZA-induced ICP cases, patient age was categorized into 10-year intervals and body weight into 10-kg intervals (Table 1). Reporting countries were grouped as Europe, Asia, United States, Oceania, or Not Specified. Clinical Outcomes were classified as either hospitalized or other outcomes, and reporting years were grouped into two-year intervals.

**TABLE 1:** Clinical characteristics of ICP cases associated with AZA use.

Charateristics	ICP induced by AZA
Gender, n (%)	
Female	67 (97.1)
Not specified	2 (2.9)
Age (years), n (%)	
20 ≤ and <30	16 (23.19)
30 ≤ and <40	36 (52.17)
≥40	6 (8.7)
Not specified	11 (15.94)
Weight (kg), n (%)	
50≤ and <60	1 (1.45)
60≤ and <70	3 (4.35)
Not specified	65 (94.2)
Report countries, n (%)	
US	47 (68.12)
Europe	11 (15.94)
Asia	6 (8.7)
Oceania	3 (4.35)
Not specified	2 (2.9)
Outcome, n (%)	
Hospitalized	9 (12.33)
Other outcomes	64 (87.67)
Reporting year, n (%)	
1968–2015	1 (1.45)
2016–2017	1 (1.45)
2018–2019	3 (4.35)
2020–2021	45 (65.22)
2022–2023	11 (15.94)
2024 Q2	8 (11.59)

n, number of reporting cases, intrahepatic cholestasis of pregnancy, ICP; azathioprine, AZA.

Case identification was based on preferred terms (PTs) in MedDRA. ICP-related PTs were selected from categories within the same System Organ Class (SOC: hepatobiliary disorders) as ICP. Representative PTs included Hepatitis, Drug-induced Liver Injury, Hepatic Cirrhosis, Liver Injury, Cholestasis, Jaundice, Non-alcoholic Fatty Liver, Cholestasis of Pregnancy, Nodular Regenerative Hyperplasia, and Foetor Hepaticus, which reflect the major hepatobiliary manifestations relevant to ICP. A complete list of all PTs used in this classification is provided in Supplementary Table S1.

Duplicate reports were removed using a two-step procedure. First, deduplication was performed by sorting and comparing records across seven clinical variables active ingredient, indication, reported PT, patient sex, event date, age, and reporting country. Entries matching across all variables were considered duplicates and removed. Second, a manual review was performed to identify potential residual duplicates, particularly cases in which the same patient appeared to have been reported more than once with slightly differing details. These records were further compared based on demographics, event timing, and AE descriptions to distinguish true duplicates from unique cases. After deduplication, the dataset was filtered to include only female patients who had received AZA.

To further refine case selection, pregnant women were identified through pregnancy-related PTs (pregnancy, first trimester pregnancy, second trimester pregnancy, third trimester pregnancy, and exposure during pregnancy) as well as reporter-provided pregnancy information, ensuring accurate classification of pregnancy-associated events. In addition, subgrouping by clinical indication (e.g., Crohn’s disease, ulcerative colitis, systemic lupus erythematosus) was performed to evaluate whether safety signals differed across relevant autoimmune conditions.

### Statistical framework for signal detection

#### Disproportionality analysis

The primary aim of this pharmacovigilance study was to evaluate the potential association between AZA and ICP using multiple disproportionality methods. Three complementary signal detection approaches were applied to enhance robustness and reduce the risk of false-positive findings [22, 23]. First, the Reporting Odds Ratio (ROR) was calculated by comparing the frequency of AZA–ICP reports with all other drug–event pairs in FAERS. RORs were computed using MedDRA PTs identified as the primary suspected AEs of AZA. A disproportionality signal was defined when the lower bound of the 95% confidence interval (CI) exceeded 1.0, and at least three unique cases remained after deduplication. Second, the Information Component (IC) from the Bayesian Confidence Propagation Neural Network (BCPNN) was generated to provide an additional Bayesian assessment of disproportionate reporting. A signal was considered present when IC025 (the lower 95% CI bound) exceeded zero. Third, a Bayesian shrinkage method based on the Multi-Item Gamma Poisson Shrinker (MGPS) algorithm was used to calculate the Empirical Bayes Geometric Mean (EBGM), and a positive Bayesian signal was defined as EB05 > 2, where EB05 represents the lower 90% confidence bound [24].

#### Subgroup analyses in pregnancy and autoimmune indications

To further explore population-specific safety signals, subgroup analyses were performed in pregnant women and in patients with autoimmune diseases commonly treated with AZA. First, a broad ICP-coded set included all AZA-related reports in which the adverse event PT explicitly contained “intrahepatic cholestasis of pregnancy,” regardless of whether pregnancy status was independently verified. This set was used for the primary disproportionality screen. Second, a pregnancy-verified subset consisted of ICP reports in which pregnancy was independently confirmed through pregnancy-related PTs or FAERS reporter fields. Pregnancy-specific interpretations were based on this verified subset. Three clinically relevant subgroups were defined:

- Group 1: Female patients administered AZA (overall female exposure).
- Group 2: Pregnant women receiving AZA, identified using pregnancy-related PTs and reporter confirmation.
- Group 3: Female patients with autoimmune indications, including CD, UC, or SLE.

For subgroups with a high number of reported PTs, only the top 20 PTs were summarized in the main figures, ranked by descending ROR, while complementary Bayesian measures (IC and EBGM) were additionally provided in the Supplementary Material to support the robustness of the detected signals.

To ensure transparency in how ICP-related hepatobiliary events were identified in pregnant women, the specific PTs used in this classification are listed below, grouped by their respective SOCs:

• Hepatobiliary Disorders: Representative PTs included Hepatic Cirrhosis, Drug-induced Liver Injury, Hepatitis, Liver Injury, ICP, Cholestasis, Liver Disorder, and Jaundice.
• Investigations: Representative PTs included Liver Function Test Increased, Hepatic Enzyme Increased, Liver Function Test Abnormal, Blood Bilirubin Increased, Aspartate Aminotransferase Increased, and Alanine Aminotransferase Increased.

#### Comparative risk assessment with other ICP-inducing drugs

**FIGURE 1:**
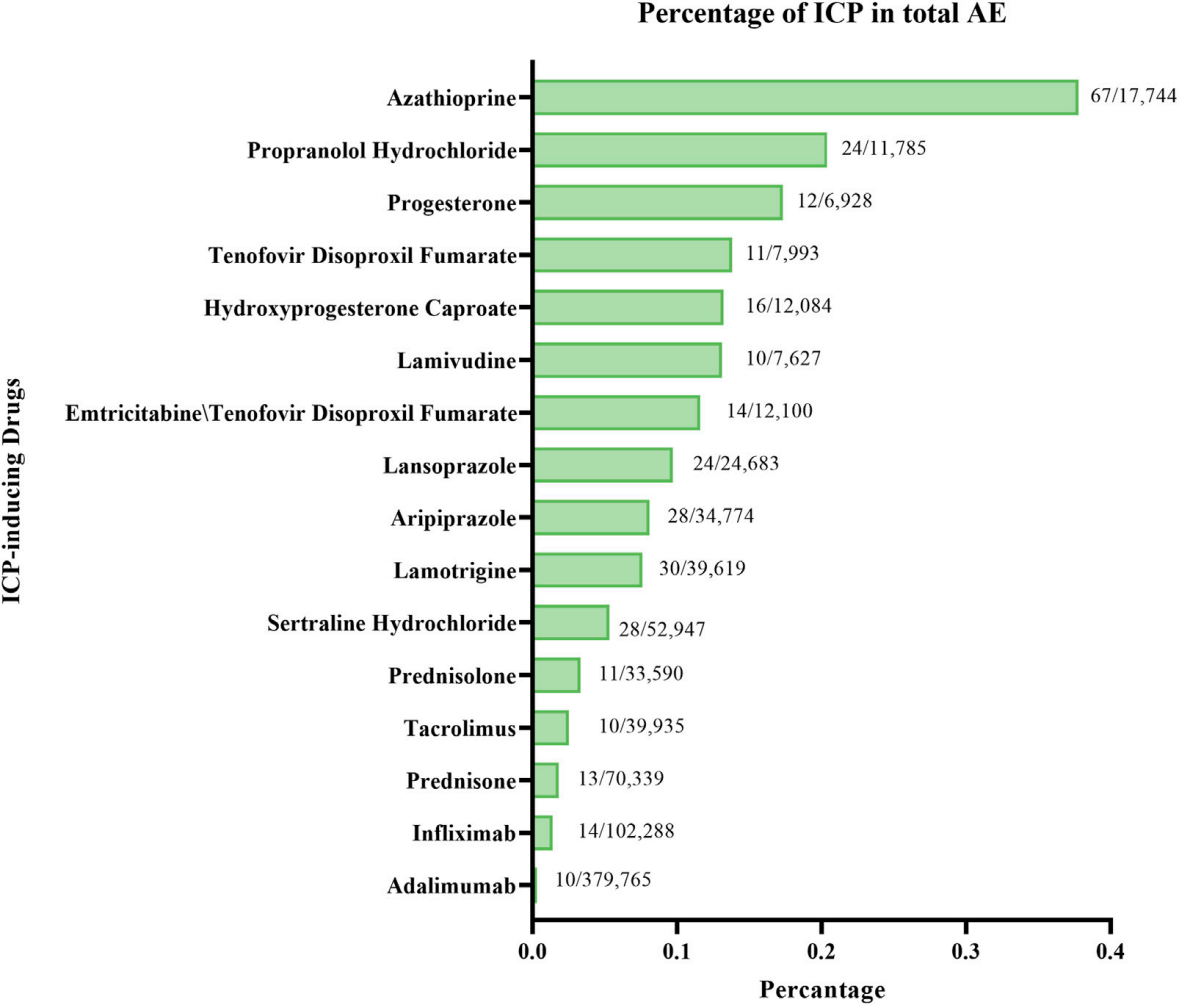
Distribution of ICP reports for AZA and other ICP-inducing drugs.

A comparative disproportionality analysis was conducted to contextualize the risk of ICP associated with AZA relative to other medications previously reported to induce ICP. In this analysis, we computed ROR, IC, and EBGM for AZA and compared them with those of established ICP-inducing drugs. In addition, the proportion of ICP-related reports attributed to AZA was compared with the corresponding proportions for these comparator drugs (Figure 1).

#### Software, reporting standards, and ethical considerations

All statistical analyses were performed using Microsoft Excel 2019 (Microsoft Corp., Redmond, WA, USA) and GraphPad Prism version 8 (GraphPad Software, San Diego, CA, USA). Graphical visualizations were generated using GraphPad Prism.

This study did not require institutional review board approval because it was based on de-identified, voluntarily submitted, publicly available reports. To ensure transparency and reproducibility, the study adhered to the REporting of A Disproportionality Analysis for DrUg Safety Signal Detection Using Individual Case Safety Reports in PharmacoVigilance (READUS-PV) criteria [25, 26].

## Results

### Clinical profile of azathioprine-associated ICP cases

**FIGURE 2:**
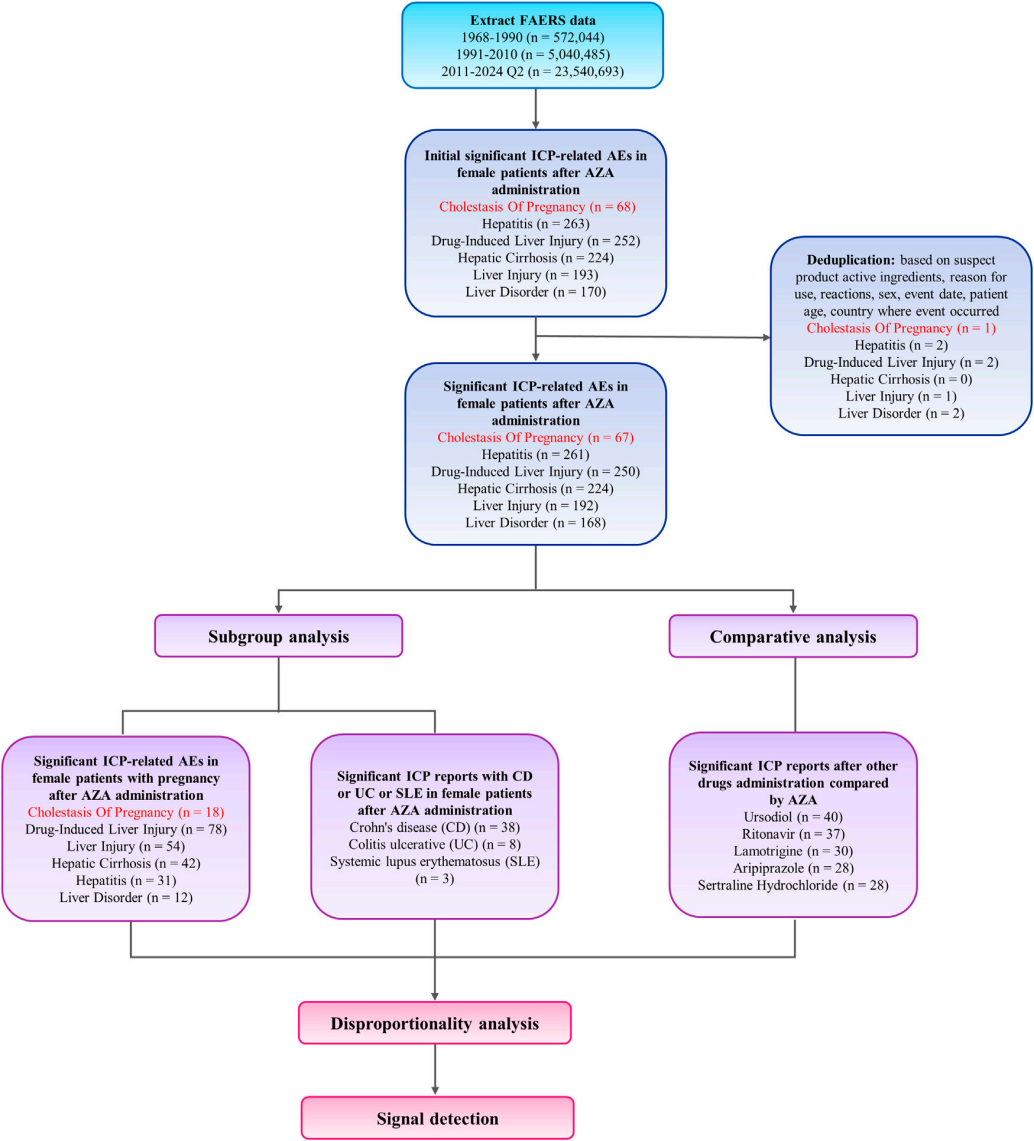
Flowchart of case identification, deduplication, and data processing steps.

The analysis identified 35,576 adverse event reports related to AZA in FAERS database, among which 67 reports were coded with the preferred term “intrahepatic cholestasis of pregnancy” (ICP) (Table 1; Figure 2). Of these, 18 had independent confirmation of pregnancy through pregnancy-related preferred terms or reporter fields, and this pregnancy-verified subset was used for pregnancy-specific analyses. The age group 30–39 years accounted for the highest proportion of ICP cases (52.17%). In terms of geography, US represented the largest proportion of ICP reports (68.12%). In the outcome category, 9 cases (12.33%) were reported as hospitalized and 64 cases (87.67%) were classified under other outcomes. Some reports listed more than one outcome for the same case, so the total number of outcomes (n = 73) is higher than the number of ICP cases. Regarding reporting year, the period 2020–2021 showed the highest reporting frequency (65.22%). Only one ICP case was reported before 2016; therefore, all reports from 1968 to 2015 were grouped into a single category to avoid sparse data, while subsequent years were grouped in 2-year intervals due to the increase in reporting volume (Table 1).

### Disproportionality signals of ICP-related preferred terms in women exposed to azathioprine

**FIGURE 3:**
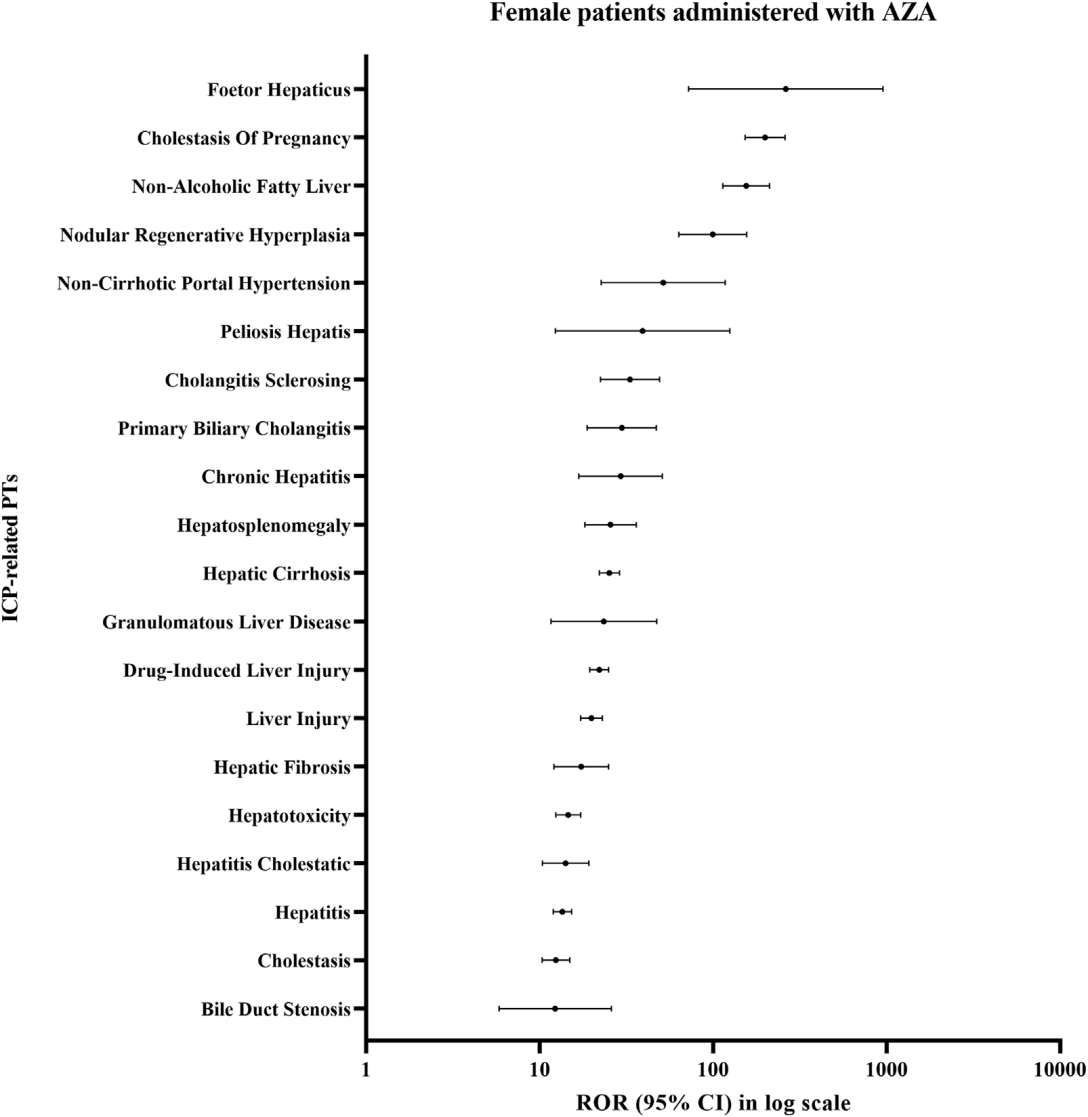
Disproportionality signals for ICP-related PTs in female patients treated with AZA.

In the signals of disproportionate reporting analysis of ICP-related PTs among female patients administered AZA, the strongest signals were observed for ICP (ROR025 = 153; IC025 = 5.8; EBGM05 = 131.6), Non-Alcoholic Fatty Liver (ROR025 = 113.69; IC025 = 5.31; EBGM05 = 115.31), Foetor Hepaticus (ROR025 = 72.28; IC025 = 0.7; EBGM05 = 182.72), and Nodular Regenerative Hyperplasia (ROR025 = 63.33; IC025 = 4.14; EBGM05 = 75.76) (Figure 3; Supplementary Table S1). Notably, the lower bound of the 95% CI for the ROR exceeded 100 for ICP and Non-Alcoholic Fatty Liver, indicating robust statistical signals.

### Pregnancy-specific safety signals: ICP and hepatobiliary events in women receiving azathioprine

**FIGURE 4:**
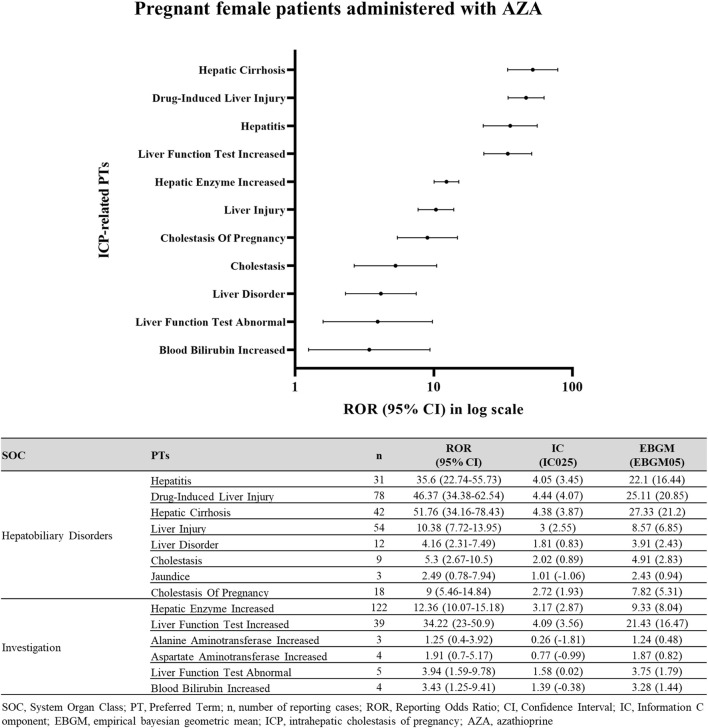
Disproportionality signals for ICP-related events in pregnant women exposed to AZA. Disproportionality analysis of ICP reports in pregnant female patients treated with AZA.

Among pregnant women exposed to AZA, ICP-related PTs were identified from the SOCs Hepatobiliary Disorders and Investigations (Figure 4). Pregnant women in this analysis were identified through pregnancy-related preferred terms or reporter fields, comprising a pregnancy-verified subset of 18 cases.

In this subgroup, ICP demonstrated a significant signal of disproportionate reporting (ROR025 = 5.46; IC025 = 1.93; EBGM05 = 5.31). Within the Hepatobiliary Disorders SOC, the lower 95% CI limits for Hepatic Cirrhosis (ROR025 = 34.16; IC025 = 3.87; EBGM05 = 21.2), Drug-induced Liver Injury (ROR025 = 34.38; IC025 = 4.07; EBGM05 = 20.85), Hepatitis (ROR025 = 22.74; IC025 = 3.45; EBGM05 = 16.44), Liver Injury (ROR025 = 7.72; IC025 = 2.55; EBGM05 = 6.85), and Cholestasis (ROR025 = 2.67; IC025 = 0.89; EBGM05 = 2.83) all exceeded 1.0, confirming significant signals. Similarly, in Investigations, Liver Function Test Increased (ROR025 = 23.0; IC025 = 3.56; EBGM05 = 16.47), and Hepatic Enzyme Increased (ROR025 = 10.07; IC025 = 2.87; EBGM05 = 8.04) met signal detection criteria.

### Subgroup disproportionality in autoimmune diseases (CD, UC, SLE)

**FIGURE 5:**
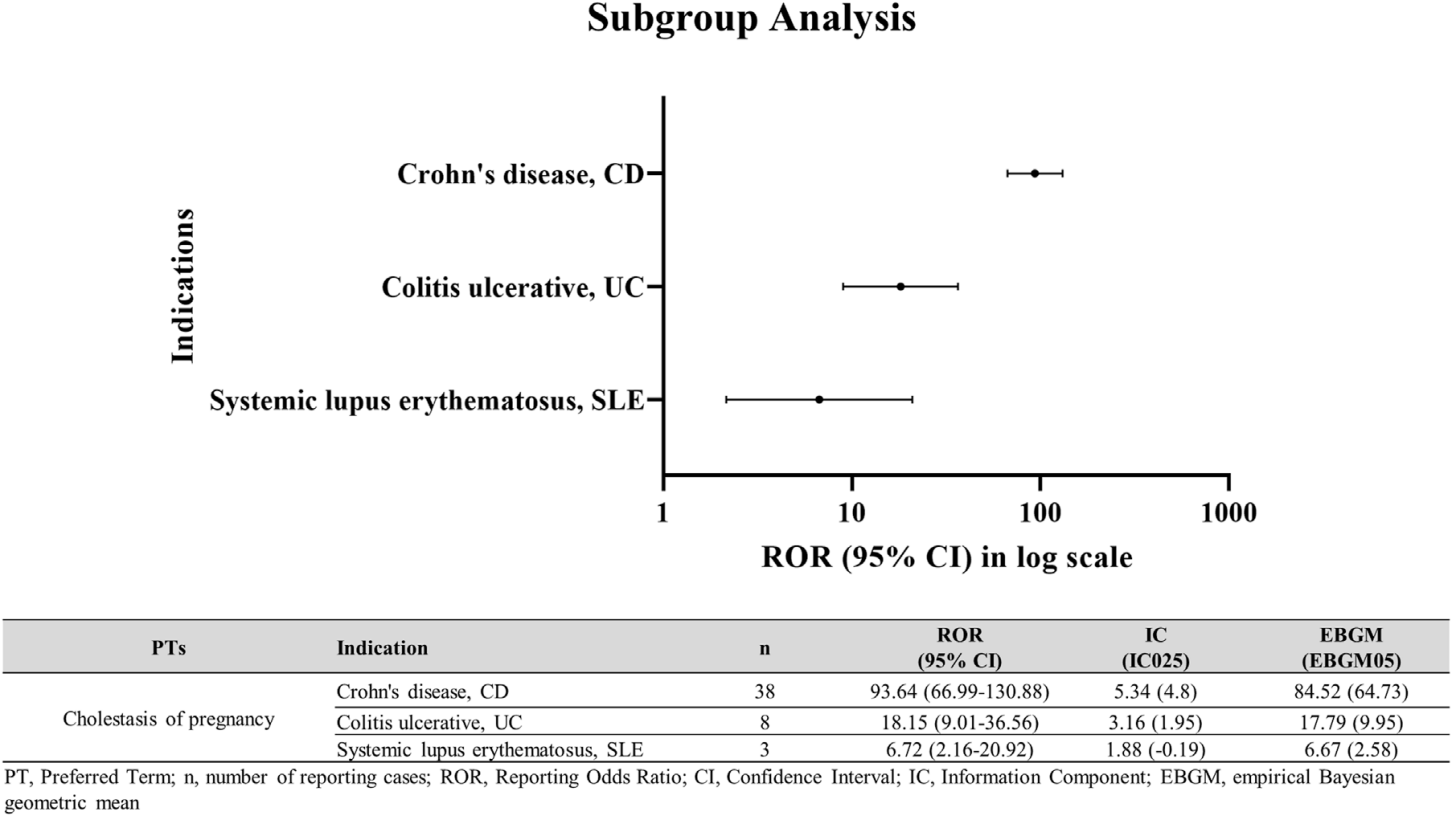
ICP disproportionality signals in autoimmune disease subgroups among AZA users. ICP in female patients with CD, UC, or SLE treated with AZA.

In subgroup analysis, AZA use in patients with underlying autoimmune diseases was significantly associated with ICP (Figure 5). Specifically, signals of disproportionate reporting were observed in Crohn’s disease (ROR025 = 66.99; IC025 = 4.8; EBGM05 = 64.73) and Colitis ulcerative (ROR025 = 9.01; IC025 = 1.95; EBGM05 = 9.95).

### Comparative risk assessment: azathioprine versus other ICP-inducing drugs

**FIGURE 6:**
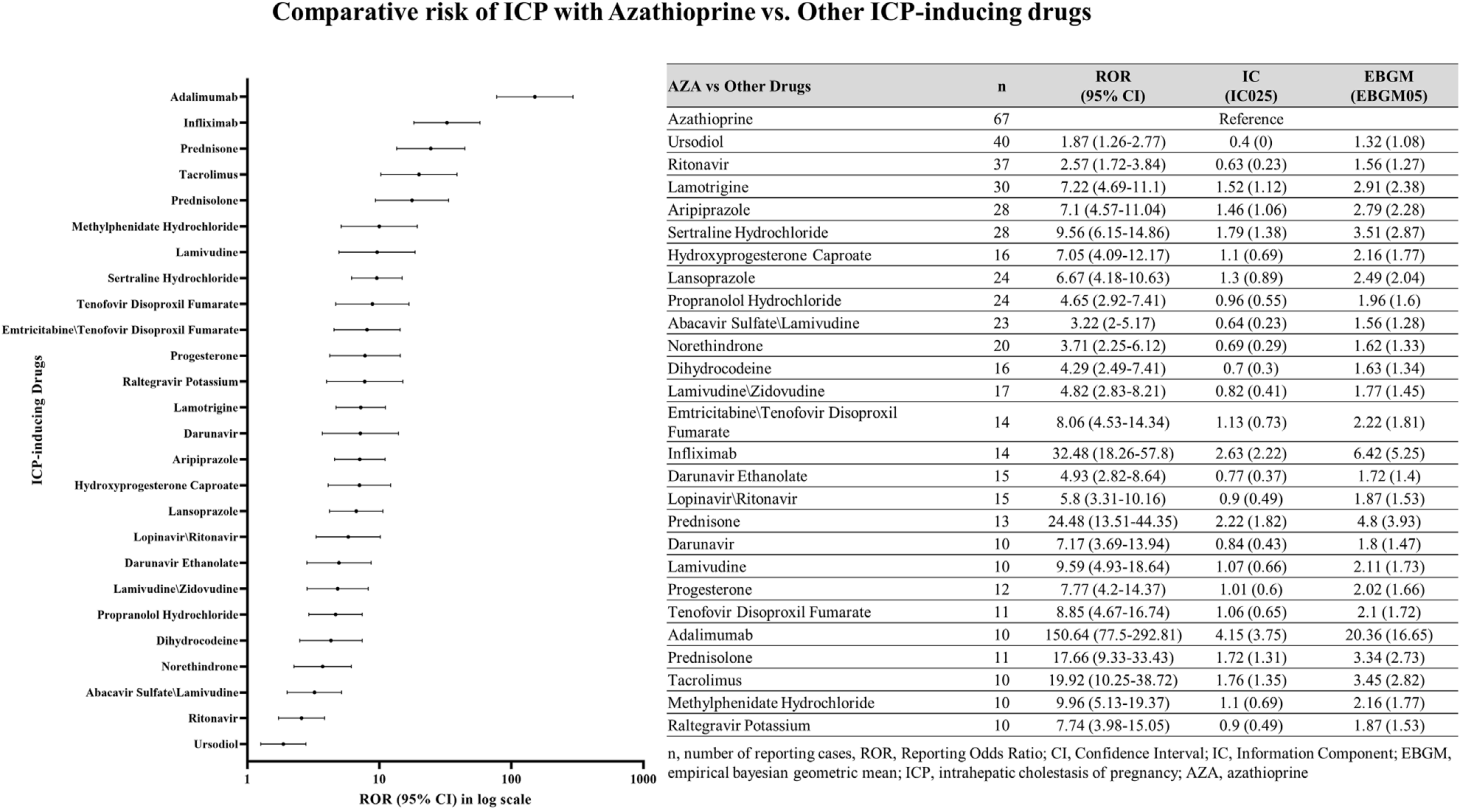
Comparative analysis of azathioprine and other ICP-inducing drugs. Comparison of ICP case reports in female patients between azathioprine and other ICP-related drugs. n, number of reporting cases; ROR, Reporting Odds Ratio; CI, Confidence Interval; intrahepatic cholestasis of pregnancy, ICP; azathioprine, AZA.

Comparative disproportionality analysis revealed that AZA was associated with a markedly elevated reporting signal of ICP compared with other ICP-inducing drugs (Figure 6). The strongest signals were observed for Adalimumab (ROR025 = 77.5; IC025 = 3.75; EBGM05 = 16.65), Infliximab (ROR025 = 18.26; IC025 = 2.22; EBGM05 = 5.25), Prednisone (ROR025 = 13.51; IC025 = 1.82; EBGM05 = 3.93), and Tacrolimus (ROR025 = 10.25; IC025 = 1.35; EBGM05 = 2.82).

Among all ICP-inducing drugs analyzed, AZA accounted for the highest proportion of ICP reports (67 cases; 0.38%, Figure 6). For comparison, other drugs with relatively high percentages of ICP reports included Propranolol Hydrochloride (24 cases; 0.20%), Progesterone (12 cases; 0.17%), Tenofovir Disoproxil Fumarate (11 cases; 0.14%), and Hydroxyprogesterone Caproate (16 cases; 0.13%). Nevertheless, AZA demonstrated the most prominent disproportionality, both in terms of absolute number and relative percentage of ICP cases.

## Discussion

This study suggests a significant association between AZA use and ICP, supporting the need for heightened clinical monitoring and risk management when prescribing AZA to pregnant patients. Our analysis revealed a markedly elevated ROR for ICP with AZA (ROR025 = 153.0; IC025 = 5.8; EBGM05 = 144.37), and pregnancy-specific subgroup analyses further identified significant signals, including ICP itself (ROR025 = 5.46; IC025 = 1.93; EBGM05 = 5.31) and hepatobiliary events such as drug-induced liver injury (ROR025 = 34.38; IC025 = 4.07; EBGM05 = 20.85). Additional subgroup analyses identified significant signals in patients with Crohn’s disease (ROR025 = 66.99; IC025 = 4.8; EBGM05 = 64.73), and Colitis ulcerative (ROR025 = 9.01; IC025 = 1.95; EBGM05 = 9.95). We also observed that the proportion of ICP reports for AZA (67 cases among 17,744 reports) was higher than that of other ICP-inducing drugs, approximately two-fold greater in relative percentage. To our knowledge, this is the first quantitative disproportionality analysis specifically evaluating AZA-associated ICP. By combining subgroup and comparative analyses, we attempted to strengthen the reliability of the signal and provide a more comprehensive understanding of this association. Importantly, pregnancy-specific interpretations in this study are based on the pregnancy-verified subset (n = 18), whereas the broader disproportionality signal reflects the full ICP-coded set (n = 67). This distinction should be considered when interpreting the findings for pregnant women.

We hypothesize that several factors may contribute to this phenomenon. One proposed mechanism involves pregnancy-related hormonal modulation of thiopurine metabolism. During pregnancy, changes in enzyme activity, including thiopurine S-methyltransferase, may alter the balance of AZA metabolism toward 6-mercaptopurine (6-MP), thereby increasing the generation of hepatotoxic metabolites [20, 27]. This metabolic diversion could amplify cholestatic effects in susceptible individuals. Additionally, AZA has been documented to cause transient elevations in liver enzyme levels and biological cholestasis [20]. Consistent with this, a French case series reported six women with IBD on AZA who developed atypical and more severe ICP earlier in pregnancy [20, 28]. In these patients, bile acid levels were markedly elevated despite normal liver enzymes, and abnormalities persisted despite UDCA therapy but resolved upon AZA discontinuation [28]. Such clinical evidence supports a potential causal role of AZA in exacerbating ICP.

Beyond individual case descriptions, the documented patterns of AZA-related hepatotoxicity offer mechanistic support for its potential to contribute to cholestatic conditions such as ICP. Early in therapy, elevations in aminotransferases have been linked to higher levels of methyl-mercaptopurine, a metabolite formed during AZA biotransformation and known to exert direct toxic effects on hepatocytes [29, 30]. AZA can also induce an acute cholestatic injury, typically presenting within 2–12 months and characterized histologically by intrahepatic cholestasis with focal hepatocellular necrosis and scant inflammation. This “bland cholestasis” pattern described as similar to the cholestasis observed with estrogens suggests impaired bile flow rather than immune-mediated hepatitis. Chronic thiopurine exposure has also been associated with sinusoidal dilation, portal venopathy, and nodular regenerative hyperplasia, reflecting structural disturbances that may interfere with biliary drainage. Rarely, prolonged cholestasis or vanishing bile duct syndrome has been described, and long-term thiopurine use has been associated with hepatocellular carcinoma and hepatosplenic T-cell lymphoma [31]. Considering these mechanisms and disease features together offers a clear biological explanation for the strong cholestasis risk observed. This strongly suggests that AZA could make cholestasis worse, especially during pregnancy.

ICP itself is a multifactorial disorder in which several converging risk factors impair bile acid homeostasis [28, 32]. Genetic variants in hepatobiliary transporters and nuclear receptors (e.g., ABCB4/MDR3, ABCB11/BSEP, ABCC2, NR1H4/FXR) predispose individuals to defective bile secretion and intrahepatic bile acid accumulation [32, 33]. Pregnancy-specific hormonal changes, particularly elevated sulfated progesterone metabolites (e.g., epiallopregnanolone sulfate) and estradiol, can downregulate BSEP expression and attenuate FXR signaling, thereby reducing bile acid clearance. In parallel, an imbalance in maternal immune responses, including increased IL-6, IL-12, IL-17, and TNF-α and reduced IL-4, may exacerbate hepatocellular injury [32, 34]. The convergence of these genetic, hormonal, and immunological influences with AZA’s hepatotoxic potential offers a plausible biological basis for the disproportionately high ROR for ICP observed in our analysis.

Beyond these biological mechanisms, disease-specific clinical and therapeutic factors may further explain the gradient in signal intensity observed across CD, UC, and SLE. Thiopurine exposure patterns during pregnancy differ across these conditions: CD carries a higher risk of flare, leading most patients to maintain thiopurine therapy throughout gestation, whereas UC is more often managed with lower or intermittent exposure. Real-world pregnancy registry data reinforce this difference. In the large prospective PIANO cohort, 41% of women with UC received no thiopurine or biologic therapy during pregnancy, compared with only 16% of those with CD [35]. Pregnant women with CD also demonstrated higher use of biologic-thiopurine combination therapy (18% vs. 11% in UC) and greater reliance on sustained immunomodulator treatment, resulting in markedly higher cumulative thiopurine exposure. In contrast, treatment strategies for SLE pregnancies differ fundamentally from those used in IBD. Management generally centers on hydroxychloroquine and low-dose corticosteroids, and when azathioprine is prescribed, it is typically administered at lower doses consistent with rheumatology guidelines recommending ≤2 mg/kg/day [18, 36, 37]. As a result, cumulative thiopurine exposure in SLE is considerably lower than in CD or UC. It is also important to acknowledge that the SLE subgroup in our dataset comprised only three ICP cases, raising the possibility that the weaker signal observed (ROR025 = 2.16; IC025 = −0.19; EBGM05 = 2.58) may reflect the small number of available reports, rather than indicating that no association exists.

This study has several limitations. First, inherent bias of disproportionality analyses and spontaneous reporting systems such as FAERS apply to this study. Underreporting and reporting biases are unavoidable, and although extensive deduplication was performed, some residual duplicates may remain. Moreover, the association between azathioprine and ICP observed in this analysis cannot be interpreted as causal. Disproportionality analyses do not allow for estimation of incidence, prevalence, or comparative risk due to the absence of reliable drug utilization data and background exposure rates in the FAERS database [38]. Given the observational design and reliance on voluntary spontaneous reports, the findings should be considered hypothesis-generating, and not interpreted as evidence of causality or as a quantification of absolute or comparative risk. Second, temporal bias may also have affected the observed disproportionality signal, as reporting patterns related to azathioprine or intrahepatic cholestasis of pregnancy may vary over time in response to regulatory alerts, increased clinical awareness, or media coverage. Third, potential confounding factors such as differences in disease severity, pregnancy status, and concomitant immunosuppressive therapies may have influenced the reporting patterns. In addition, indication bias and cumulative exposure should be considered, as azathioprine has been widely used for decades in chronic autoimmune conditions like CD, UC and SLE, which themselves may increase the risk of ICP. These factors make it difficult to determine whether the observed signal is attributable to azathioprine, the underlying disease, or their interaction. Finally, the mechanistic explanation presented in this study is based on a synthesis of existing literature and should be regarded as hypothesis-generating rather than conclusive, as this study did not directly assess biological mechanisms. Despite these limitations, we mitigated bias by conducting subgroup and comparative analyses, which consistently demonstrated elevated disproportionality signals for AZA-associated ICP.

## Conclusion

In conclusion, this pharmacovigilance analysis identifies a novel disproportionality signal suggesting a possible association between AZA exposure and reports of intrahepatic cholestasis of pregnancy. Given the clinical importance of both disease control and maternal–fetal safety, clinicians should weigh the benefits of AZA therapy against the potential risk of ICP and consider enhanced monitoring strategies, especially in women of reproductive age with autoimmune diseases. Future prospective studies and mechanistic investigations are warranted to validate these findings and to clarify the underlying biological pathways.

## Data Availability

The original contributions presented in the study are included in the article/Supplementary Material, further inquiries can be directed to the corresponding author.
