# Supplementary material for: Intrahepatic cholestasis of pregnancy associated with azathioprine: first quantitative disproportionality analysis using the FDA adverse event reporting system
Source: J Pharm Pharm Sci. 2025 Dec 16;28:15527. doi: 10.3389/jpps.2025.15527 (PMC12748001; doi:10.3389/jpps.2025.15527)
Supplement: Supplementary file 1 [file Table1.docx]

| **Supplementary Table 1.** Disproportionality analysis of ICP reports in female patients treated with AZA | | | | | |
| --- | --- | --- | --- | --- | --- |
| **SOC** | **PTs** | **n** | **ROR (95% CI)** | **IC (IC025)** | **EBGM (EBGM05)** |
| Hepatobiliary Disorders | Hepatitis | 261 | 13.53 (11.97-15.31) | 3.69 (3.48) | 13.16 (8.42) |
|  | Drug-Induced Liver Injury | 250 | 22.04 (19.43-25.01) | 4.35 (4.14) | 21.25 (14.97) |
|  | Hepatic Cirrhosis | 224 | 25.24 (22.08-28.85) | 4.53 (4.31) | 24.27 (17.49) |
|  | Liver Injury | 192 | 19.88 (17.22-22.95) | 4.2 (3.96) | 19.26 (13.32) |
|  | Liver Disorder | 168 | 7.4 (6.35-8.62) | 2.84 (2.58) | 7.29 (3.98) |
|  | Hepatotoxicity | 144 | 14.61 (12.38-17.23) | 3.77 (3.5) | 14.28 (9.28) |
|  | Cholestasis | 116 | 12.41 (10.33-14.91) | 3.54 (3.23) | 12.18 (7.63) |
|  | Jaundice | 108 | 5.66 (4.69-6.85) | 2.46 (2.14) | 5.61 (2.81) |
|  | Hepatic Function Abnormal | 97 | 3.35 (2.74-4.09) | 1.72 (1.38) | 3.33 (1.36) |
|  | Hepatic Failure | 69 | 4.04 (3.19-5.13) | 1.98 (1.58) | 4.02 (1.77) |
|  | Cholestasis Of Pregnancy | 67 | 199.56 (153-260.29) | 6.21 (5.8) | 162.2 (144.37) |
|  | Cholelithiasis | 50 | 2.11 (1.6-2.78) | 1.06 (0.59) | 2.1 (0.68) |
|  | Non-Alcoholic Fatty Liver | 47 | 155.08 (113.69-211.54) | 5.79 (5.31) | 131.6 (115.31) |
|  | Autoimmune Hepatitis | 46 | 11.51 (8.6-15.39) | 3.35 (2.86) | 11.34 (6.98) |
|  | Hepatitis Cholestatic | 41 | 14.12 (10.37-19.23) | 3.59 (3.07) | 13.88 (8.96) |
|  | Hepatic Steatosis | 44 | 3.75 (2.79-5.04) | 1.86 (1.35) | 3.73 (1.6) |
|  | Hepatic Cytolysis | 35 | 3.11 (2.23-4.34) | 1.59 (1.03) | 3.1 (1.22) |
|  | Hepatosplenomegaly | 34 | 25.62 (18.21-36.04) | 4.21 (3.64) | 24.87 (17.97) |
|  | Hepatic Necrosis | 31 | 8.04 (5.65-11.46) | 2.84 (2.24) | 7.97 (4.46) |
|  | Hepatic Fibrosis | 30 | 17.37 (12.1-24.95) | 3.75 (3.14) | 17.03 (11.48) |
|  | Acute Hepatic Failure | 28 | 4.82 (3.33-7) | 2.17 (1.54) | 4.8 (2.27) |
|  | Hyperbilirubinaemia | 27 | 3.87 (2.65-5.66) | 1.87 (1.23) | 3.86 (1.67) |
|  | Hepatocellular Injury | 26 | 3.66 (2.49-5.38) | 1.8 (1.14) | 3.64 (1.54) |
|  | Cholangitis Sclerosing | 26 | 33.13 (22.39-49.02) | 4.33 (3.68) | 31.91 (23.98) |
|  | Hypertransaminasaemia | 24 | 5.81 (3.89-8.68) | 2.39 (1.71) | 5.77 (2.92) |
|  | Nodular Regenerative Hyperplasia | 21 | 99.48 (63.33-156.27) | 4.87 (4.14) | 89.34 (75.76) |
|  | Jaundice Cholestatic | 20 | 5.18 (3.34-8.04) | 2.23 (1.48) | 5.15 (2.5) |
|  | Hepatomegaly | 20 | 3.41 (2.2-5.29) | 1.68 (0.93) | 3.4 (1.39) |
|  | Portal Hypertension | 19 | 14.48 (9.2-22.79) | 3.41 (2.64) | 14.24 (9.24) |
|  | Primary Biliary Cholangitis | 19 | 29.73 (18.82-46.98) | 4.07 (3.3) | 28.76 (21.27) |
|  | Cholangitis | 18 | 7.68 (4.83-12.22) | 2.69 (1.9) | 7.62 (4.21) |
|  | Hepatitis Acute | 16 | 4.82 (2.95-7.88) | 2.1 (1.26) | 4.8 (2.27) |
|  | Cholecystitis | 15 | 1.91 (1.15-3.17) | 0.89 (0.02) | 1.91 (0.58) |
|  | Cholecystitis Acute | 14 | 4.12 (2.44-6.97) | 1.89 (0.99) | 4.1 (1.83) |
|  | Venoocclusive Liver Disease | 14 | 10 (5.91-16.95) | 2.92 (2.02) | 9.9 (5.88) |
|  | Hepatitis Toxic | 14 | 9.91 (5.85-16.78) | 2.91 (2.01) | 9.8 (5.81) |
|  | Chronic Hepatitis | 13 | 29.28 (16.84-50.88) | 3.82 (2.88) | 28.34 (20.91) |
|  | Gallbladder Disorder | 11 | 0.8 (0.44-1.44) | -0.31 (-1.34) | 0.8 (0.13) |
|  | Hepatic Mass | 10 | 8.09 (4.34-15.09) | 2.59 (1.51) | 8.03 (4.5) |
|  | Biliary Tract Disorder | 9 | 9.65 (5-18.61) | 2.72 (1.58) | 9.55 (5.62) |
|  | Hepatic Cyst | 8 | 3.45 (1.72-6.9) | 1.59 (0.38) | 3.44 (1.42) |
|  | Biliary Obstruction | 8 | 4.95 (2.47-9.91) | 2 (0.79) | 4.92 (2.35) |
|  | Granulomatous Liver Disease | 8 | 23.43 (11.61-47.3) | 3.32 (2.11) | 22.84 (16.26) |
|  | Bile Duct Stenosis | 7 | 12.28 (5.83-25.91) | 2.8 (1.5) | 12.12 (7.58) |
|  | Congestive Hepatopathy | 7 | 7.85 (3.73-16.52) | 2.42 (1.12) | 7.79 (4.33) |
|  | Cholestatic Liver Injury | 7 | 9.34 (4.44-19.68) | 2.58 (1.28) | 9.25 (5.4) |
|  | Bile Duct Stone | 6 | 3.16 (1.42-7.03) | 1.43 (0.02) | 3.15 (1.25) |
|  | Vanishing Bile Duct Syndrome | 5 | 13.03 (5.39-31.51) | 2.63 (1.07) | 12.85 (8.15) |
|  | Non-Cirrhotic Portal Hypertension | 6 | 51.51 (22.61-117.36) | 3.38 (1.97) | 48.69 (38.71) |
|  | Ocular Icterus | 5 | 2.11 (0.88-5.07) | 0.94 (-0.63) | 2.1 (0.68) |
|  | Hepatic Lesion | 4 | 1.67 (0.63-4.46) | 0.64 (-1.13) | 1.67 (0.47) |
|  | Portal Vein Thrombosis | 4 | 2.95 (1.11-7.88) | 1.28 (-0.49) | 2.94 (1.13) |
|  | Mixed Liver Injury | 4 | 3.48 (1.31-9.3) | 1.45 (-0.32) | 3.47 (1.44) |
|  | Cholecystitis Chronic | 3 | 0.42 (0.13-1.29) | -1.13 (-3.2) | 0.42 (0.03) |
|  | Hepatic Atrophy | 3 | 9.48 (3.04-29.58) | 2.09 (0.03) | 9.39 (5.5) |
|  | Hepatitis Fulminant | 3 | 1.97 (0.63-6.12) | 0.79 (-1.28) | 1.97 (0.61) |
|  | Hepatorenal Syndrome | 3 | 2.56 (0.82-7.96) | 1.06 (-1) | 2.56 (0.92) |
|  | Hyperbilirubinaemia Neonatal | 3 | 12.81 (4.1-40.06) | 2.25 (0.18) | 12.64 (7.98) |
|  | Foetor Hepaticus | 3 | 262.65 (72.28-954.46) | 2.77 (0.7) | 202.24 (182.72) |
|  | Peliosis Hepatis | 3 | 39.2 (12.33-124.65) | 2.59 (0.52) | 37.56 (28.88) |
| SOC, System Organ Class; PT, Preferred Term; n, number of reporting cases; ROR, Reporting Odds Ratio; CI, Confidence Interval; IC, Information Component; EBGM, empirical bayesian geometric mean; ICP, intrahepatic cholestasis of pregnancy; AZA, azathioprine | | | | | |
